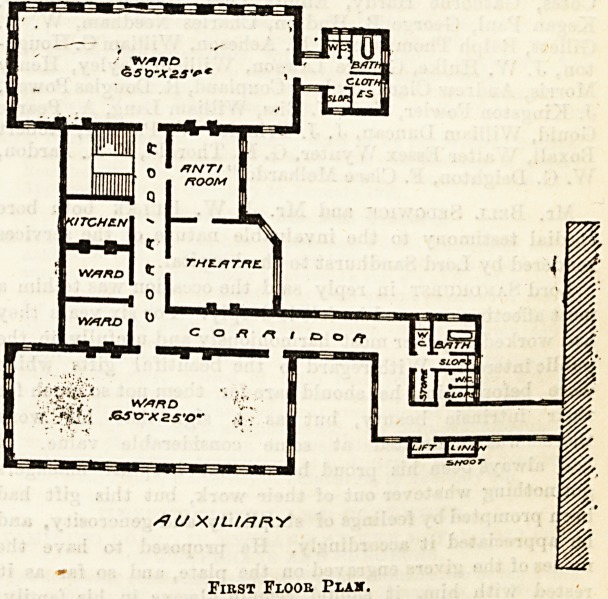# Hospital Construction

**Published:** 1895-01-19

**Authors:** 


### HOSPITAL CONSTRUCTION

### THE ROTUNDA HOSPITAL, DUBLIN.

The Rotunda Hospital at Dublin is the oldest lying-in hospital in the United Kingdom, and is also by far the most important one of its class. It was founded in 1745, and received a royal charter in 1756. Originally intended for the re-

Ground Floor Plan.

ception of lying-in women only, it has been added to, and its sphere of usefulness widened from time to time. It now includes, besides the parent lying-in hospital, an auxiliary hospital for the treatment of diseases peculiar to women, an extern maternity department, a dispensary for out-patients, an educational branch for men, and one for women. The plans we publish to-day show a new wing containing wards for the reception of female patients suffering from diseases special to their sex. The building is three storeys in height, and is connected on all floors with the original hospital by a corridor. There is a separate entrance approached by a gate-way and carriage drive from Granby Row. In arranging his building the architect (Mr. Albert E. Murray, A.R.H.A.) would seem to have first fixed, or had fixed for him, two large wards on the first floor, one at each end of the building, with small wards, a stair case, and a theatre interposed. These wards are each 65 feet by 25 feet, and have windows on one side and at each end, the other side in one ward having two windows, and in the other ward one window. This arrangement is a most unfortunate one, as the ventilation of the wards can hardly fail to be unsatisfactory; in winter, when the fires are alight, there will be some possibility of through currents of air; but in summer there can be little hope of effective ventilation. The positions of the fire-places are by no means the best possible for distributing the warmth generally over the wards. The beds not being shown on the plan, we have no exact knowledge of the number of patients the wards are intended to hold, but we understand that the two wards accommodate together about fifty patients. The sanitary offices are, in one ward, properly disconnected by a cross-ventilated lobby; in the case of the other ward the disconnection is not quite so satisfactory, but the offices are not entered directly from the ward. The theatre is a large room with one angle canted off, apparently to secure the maximum of light obtainable, but with very unfortunate result in the small ward on the floor below. The two rooms for the doctors are under one of the large wards, and of necessity are each 25 feet in length by something like 12 feet in width—an unpleasant proportion for the purpose, which is not improved by the position of the doors. Under the other large ward are dormitories for ward-maids and servants. The second floor contains dormitories for nurses.

An apparent defect of the arrangement is the planning of the large wards, but cross-ventilation and a bed between two windows may be carried too far for patients who are really ill. The experience of the Children's Hospital, Great Ormond Street, proves this, and physicians and others who spend much time in hospitals urge that windows should not be placed too

First Floor Plan.

close together. A wider intervening wall space being a relief to the patients' eyes.

The exterior of the building shows a handsome elevation in Italian style faced with red bricks and buff terra-cotta.

## Figures and Tables

**Figure f1:**
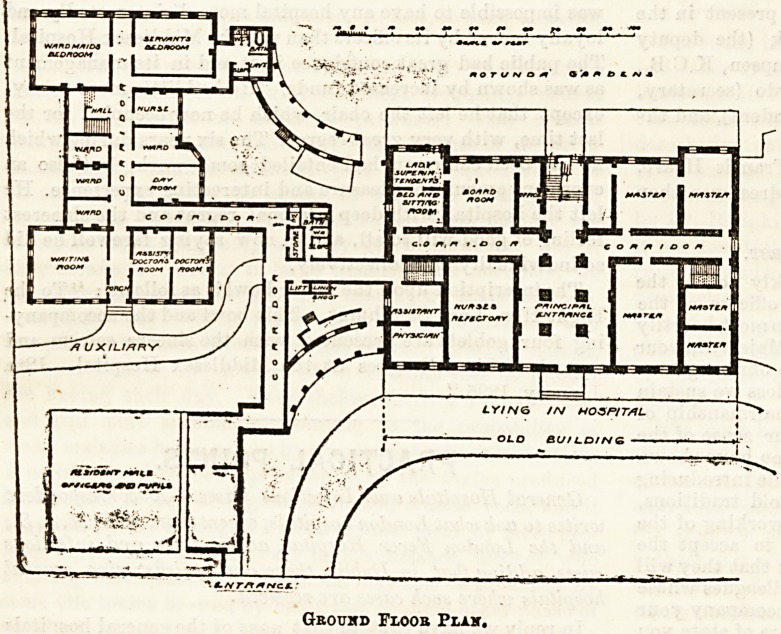


**Figure f2:**